# Outcomes by birth setting and caregiver for low risk women in Indonesia: a systematic literature review

**DOI:** 10.1186/s12978-019-0724-7

**Published:** 2019-05-28

**Authors:** Kai Hodgkin, Grace Joshy, Jenny Browne, Istri Bartini, Terence H. Hull, Kamalini Lokuge

**Affiliations:** 1 National Centre for Epidemiology & Population Health, The Australian National University, Building 62, Mills Road, Canberra, ACT 2601 Australia; 2 Midwifery, Faculty of Health, University of Canberra, Bruce, ACT 2601 Australia; 3 School of Health Sciences, Akademi Kebidanan Yogyakarta, Jl. Parangtritis Km. 6 Sewon, Yogyakarta, DIY Indonesia; 4 School of Demography College of Arts and Social Sciences, The Australian National University, 9 Fellows Road, Acton, ACT 2601 Australia

**Keywords:** Indonesia, Birth, Midwives, Homebirth, Delivery, Obstetric care, Parturition, Development, Skilled birth attendant, Midwifery

## Abstract

**Background:**

Care for women during pregnancy, labour, birth and the postpartum period is essential to reducing maternal and neonatal mortality and morbidity, however the ideal place and organisation of care provision has not been established. The World Health Organization recommends a two-tier maternity care system involving first-level care in community facilities, with backup obstetric hospital care. However, evidence from high-income countries is increasingly showing benefits for low risk women birthing outside of hospital with skilled birth assistance and access to backup care, including lower rates of intervention. Indonesia is a lower middle-income country with a network of village based midwives who attend births at homes, clinics and hospitals, and has reduced mortality rates in recent decades while maintaining largely low rates of intervention. However, the country has not met its neonatal or maternal mortality reduction goals, and it is unclear whether greater improvements could be made if all women birthed in hospital.

**Body:**

This paper reviewed the literature on birth outcomes by place of birth and/or caregiver for women considering their risk of complications in Indonesia. A systematic literature search of Pubmed, CINAHL, CENTRAL, Web of Science, Popline, WHOLIS and clinical trials registers in 2016 and updated in 2018 resulted in screening 2211 studies after removing duplicates. Twenty four studies were found to present outcomes by place of birth or caregiver and were included. The studies were varied in their findings with respect of the outcomes for women birthing at home and in hospital, with and without skilled care. The quality of most studies was rated as poor or moderate using the Effective Public Health Practice Project Quality Assessment Tool. Only one study gave an overall assessment of the risk status of the women included, making it impossible to draw conclusions about outcomes for low risk women specifically; other studies adjusted for various individual risk factors.

**Conclusion:**

From the studies in this review, it is impossible to assess the outcomes for low risk women birthing with health professionals within and outside of Indonesian hospitals. This finding is supported by reviews from other countries with developing maternity systems. Better evidence and information is needed before determinations can be made about whether attended birth outside of hospitals is a safe option for low risk women outside of high income countries.

**Electronic supplementary material:**

The online version of this article (10.1186/s12978-019-0724-7) contains supplementary material, which is available to authorized users.

## Plain English summary

Women need care by a skilled health professional during pregnancy and childbirth to identify, prevent and treat health issues for her and her baby if they should arise. We do not know if providing that care at home is as safe as in a hospital, particularly in poorer and developing countries. In wealthy countries, attended homebirth is a rare option but found to be safe if the woman choosing it is at low risk of complications and has access to a hospital if needed. In Indonesia, women birth at home, in health centres and in hospitals, and most with a health professional, unlike most lower and middle income countries, making Indonesia a good place to study the safety of birth in these different settings. This paper thoroughly searched for and summarised existing studies on this topic in Indonesia. Twenty four studies were found, most were of low quality and they found conflicting results. Some said homebirth was the safest option, others found hospital was safer. Most did not assess whether the women birthing at home were well or had complications which would have meant hospital birth was a better option for those women. We conclude that more research is needed to assess whether attended home birth is a safe option in Indonesia, particularly for low risk women.

## Background

Countries vary widely in their approaches to maternity care. In high income countries birth occurs mostly within a hospital environment with care supervision by obstetricians and midwives or nurse-midwives. Aside from a few exceptions such as The Netherlands, birth at home is often controversial, rare and not well supported by governments, insurers or private health institutions. Outside of high income countries, birth outside of hospitals is more common, and access to medical facilities and obstetricians may be restricted by a lack of many factors such as recognition of need, availability, transport, finances, or culturally or socially appropriate care [1, 2]. Care may be unavailable, or provided at home or in community health facilities by a family member, a traditional birth attendant (usually an elderly female member of the community experienced with birth but with either none or minimal medical training), a community health worker, midwife, nurse, generalist doctor or specialist obstetrician.

Ensuring that every woman has a skilled birth attendant (SBA) is a key part of international recommendations for improving maternal and neonatal outcomes. An SBA is defined as “an accredited health professional — such as a midwife, doctor or nurse — who has been educated and trained to proficiency in the skills needed to manage normal (uncomplicated) pregnancies, childbirth and the immediate postnatal period, and in the identification, management and referral of complications in women and newborns” [3]. The Millennium Development Goals defined birth with an SBA as a key indicator towards improvement in maternal health [4] and the new Sustainable Development Goals Agenda aims for universal access to sexual and reproductive health services (including skilled birth care) by 2030 [5].

There is no decisive evidence on whether birth setting – where, by whom and with what facilities - influences pregnancy outcomes for low risk women in low income countries. The World Health Organization has for over a decade advocated a two tier approach; first level care in district health centres located near to women’s homes and back-up care at centrally located tertiary referral hospitals [6]. The proposal for first-level care is that it be midwifery-led and professional yet home-like. Back-up care is recommended in all hospitals, provided by obstetricians and paediatricians and including complex interventions such as intensive care and emergency caesarean section. This strategy is based on the premise of providing skilled care to all women in a logical provider-focused framework and extrapolating positive outcomes from individual obstetric care interventions [7]. However, there is little evidence assessing the outcomes of care provision such as this [8]. An alternative option is to provide first-level care at home, with the same back-up care in tertiary referral hospitals, which is also under-researched.

In high income countries, evidence is increasingly showing benefits for women at low risk of complications who choose to birth outside of hospital and are attended by skilled carers. These women experience lower rates of vacuum or forceps birth [9, 10], caesarean section [10–12], and greater satisfaction [13–15] and breastfeeding rates [11], and their babies have fewer admissions to neonatal intensive care [9] than similar low risk women who plan hospital birth. Medical interventions such as induction of labour and caesarean section are beneficial or life-saving in some contexts, yet they can also be inappropriately or routinely used with potentially harmful outcomes [16]. Despite the lower rates of interventions, no difference in maternal or neonatal mortality rates have been observed between attended, low risk home and hospital birth in Australia [17, 18], New Zealand [12], The United Kingdom [10], the Netherlands [19] or Canada [9, 11]. Additionally, home births have been found to be more cost effective than hospital births [20, 21].

Debate still exists over the safety of planned, low risk home birth in high-income countries, though there has been no consistency of negative findings. Importantly, in a meta-analysis conducted in 2010 which included 7 studies reporting on neonatal mortality, this was found to be higher in planned home births compared with hospital births (OR 1.98, 95% CI 1.19–3.28) [22]. However, this study has been criticised for its quality and inclusion criteria [23], and when the authors excluded home births not attended by certified midwives or nurse midwives, the odds ratio was no longer significant (OR 1.57, 95% CI 0.62–3.98). The Birthplace in England Study found higher odds of negative neonatal outcomes for low risk women having their first baby in planned home births compared with obstetric units (aOR 1.75, 95% CI 1.07 to 2.86), though not for those having their second or subsequent baby [10]. While most studies have found no difference in neonatal mortality, some less serious negative findings have been seen for home births such as higher rates of admission to neonatal intensive care when compared with hospital birth [18] or high rates of intrapartum transfer, particularly for those having their first baby [10]. Though other studies have not supported these findings or have not presented outcomes separated by parity possibly due to losing statistical power with lower numbers [24]. This, along with a lack of randomised control trials and differences in health systems are among the issues that plague the debate over the quality of home birth studies and their applicability to policy and practise decisions [25].

However, the overall positive outcomes are supporting increased acceptability for home birth in many high income countries. Women in The Netherlands, New Zealand and Canada are publicly financially supported in their choice of birth setting and care provider including home birth [12, 13, 26], and almost all states and territories in Australia now have government funded home birth programs [17]. Recent policy shift in the UK encourages low risk women to birth out of hospital. The UK’s National Institute for Health and Care Excellence guideline on intrapartum care states that all women at low risk of complications should be offered the option of birthing out of hospital, and that home birth is “particularly suitable” for those birthing their second or subsequent baby [27]. Overall, in high income countries, where women have easy access to transfer to a hospital, and high quality antenatal and intrapartum care, choosing a setting other than hospital for birth results in many benefits without compromising safety to mother or baby.

The vast majority of this literature and the subsequent policy decisions about home birth focus on low risk women, though no international definition of what constitutes risk of birth complications exists. Women are, however, routinely assessed to determine their risk of complications during birth and therefore the type of care which best suits them [28]. Comparing two low risk groups of women is key to ensure data is not biased as it can be assumed that most women with ‘high-risk’ pregnancies will birth in hospitals and with obstetric care if they are available [28]. Similar risk of bias exists from not taking into consideration where a woman *plans* to birth, especially if this is different to where she actually births, though collecting this data is fraught with difficulties, contributing to reasons for a lack of studies from low resource settings.

Indonesia is potentially a useful case study to investigate whether attended low risk homebirth is safe in lower income, higher mortality contexts. Indonesia is one of the few countries which has developed their maternal health system by including efforts to provide skilled birth care in communities and homes, unlike most other countries where skilled birth care is limited to hospitals or health centres. The backbone of the Indonesian system is thousands of village based midwives who provide reproductive, maternal and child health services, often including birthing at home, or in clinics in villages [29]. These Indonesian village midwives, along with a range of community-level basic health care facilities called puskesmas (sub-district health centre – daily clinic or inpatient with basic surgery), pustu (village health centre – usually rural with daily clinic) and posyandu (usually once per month health services delivered in small/remote villages) provide public primary health care across the country [30]. Public hospitals support these centres as referral facilities in each district and province, and administration is decentralised to local governments. Many Indonesian midwives also work in their own or other private practices contributing to the large private sector in Indonesian healthcare. Around a third of hospitals are owned by private organisations, publicly owned facilities may also be privately run, and many healthcare staff work concurrently in both private practices and public facilities. This increasing privatisation contributes to significant disparities in access to health services and health outcomes between rich and poor, urban and rural [31].

The village midwife program (*bidan di desa*) was started in 1989 by the government and supported by international aid agencies. At the time, most women birthed at home with the help of a traditional birth attendant (*dukun bayi)* who had little or no health training. An increase from 13,000 midwives to over 50,000 by the end of the 1996/97 budget year providing birth assistance to 96% of villages in Indonesia [32] was achieved through government supported training and 3 year rural placement programs [33]. Unfortunately the rush resulted in compromised quality of training programs, mentoring, supervision and support once in practice, and limited access to obstetric consultation and referral [33].

Health improvements were seen for women and their children after the introduction of the village midwife program and many of the features of the program still exist, though it is unclear how much was due to the program as opposed to other factors. Maternal mortality declined over the period from a ratio of 404–600 per 100,000 live births in 1990 to 333–370 in 2000, and infant mortality declined from an estimated 68 to 32 per 1000 live births [34]. Sixty-nine percent of Indonesia’s births were attended by a midwife in 2013, most of these in a clinic, hospital or other health facility [35]. Around 30% of women birthed at home, most with a qualified midwife and many women express a preference for home birth, or a belief that hospitals are only for when illness or complications occur [34–36].

Indonesia still carries a large burden of maternal and neonatal deaths. The World Health Organization estimates Indonesia’s 2015 maternal mortality ratio at 126 per 100,000 live births [37]. The only countries in South East Asia with higher maternal mortality rates are Myanmar and Timor Leste, and Indonesia has a far larger population and therefore an overall higher number of deaths [38]. Most of Indonesia’s maternal deaths are related to hypertension and delays in accessing care; poor quality of care and lack of provisions in health services are common [34, 36].

Indonesia is now faced with the question of whether to continue with a health policy which supports midwives as first-level care in or outside of hospital, or whether the focus should be on increasing hospital birth for all women as the next step in further reducing maternal and neonatal mortality. Central to this is whether attended homebirth is safe for women in Indonesia, particularly those at low risk of complications. A policy shift has far reaching impacts including the need to review related workforce structure, guidelines, funding priorities and aid spending in Indonesia, and may provide guidance for other lower or middle income country settings.

### Aim

This review aimed to identify and review literature reporting maternal and neonatal outcomes by place of birth and/or caregiver in women with differing levels of risk of birth complications, in Indonesia.

## Methods

### Statement of the PICOS

Study population included women giving birth in Indonesia and their babies, and for whom risk factors for complications during birth were quantified. Place of birth was the main exposure assessed, with the primary comparison being between groups with different places of birth, in those low risk women who gave birth with a skilled attendant. Primary outcomes were maternal and perinatal death or serious morbidity. Other outcomes assessed included spontaneous vaginal birth, assisted birth (forceps or vacuum), caesarean section, postpartum haemorrhage, labour augmentation or induction, episiotomy, position of birth, analgesia/anaesthesia for labour or birth, known support person present for labour and birth, maternal views of care, neonatal admission to intensive care, Apgar scores, and breastfeeding rates at any point of time. These outcomes were adapted from the Cochrane Review on alternative versus conventional birth settings [39] and was decided on so as to ensure that the broad range of impacts from range of birth settings and caregivers available to women in Indonesia could be assessed. Additionally, the studies did not need to focus primarily on outcomes by place of birth, this could be a secondary outcome, with primary focus being interventions such as training or antenatal care. The search included randomised, quasi-randomised, observational and non-randomised intervention studies.

### Search strategy

The methodology of this review was developed based on the Preferred Reporting Items for Systematic Reviews and Meta-Analyses (PRISMA) guidelines [40]. No protocol was registered. A systematic search was conducted of Pubmed, CINAHL, CENTRAL, Web of Science, Popline, WHOLIS and ANZ and US clinical trials registers using keywords and MeSH terms related to birth setting and caregiver combined with the keyword ‘Indonesia’. The initial search was conducted in July 2016 and subsequently updated in November - December 2018, and included studies in both English and Indonesian language. The search terms used are available in Additional file 2: Appendix 2.

### Eligibility and exclusion criteria

Articles were restricted to 1997 or later, as this is when the majority of women in Indonesia had at least access to skilled birth assistance through the village midwife program. Articles were removed if they were duplicates, did not provide a comparison of at least one outcome (listed below) by birth setting or caregiver, and if they did not include some measure of pregnancy risk status, where risk factors for complication at birth have been identified and accounted for, either through segregation (low and high risk grouping) or through analysis (treating risk factors as potential confounders). A list of risk factors (Table 1) was extracted from the antenatal care guidelines for identification of complications in the “Pocket Book: Health Care and Referral of Women in Basic Health Facilities” by the Indonesian Ministry of Health, the World Health Organization, Indonesian Obstetric and Gynaecology Organisation and the Indonesian Midwifery Association [41].

**Table 1:** List of risk factors for complications at birth. Translated and adapted from: *Buku Saku: Pelayanan Kesehatan Ibu di Fasilitas Kesehatan Dasar dan Rujukan* (2013) Kementarian Kesehatan Republik Indonesia, World Health Organization, Perkumpulan Obstetri dan Ginekologi Indonesia, Ikatan Bidan Indonesia. *(Pocket Book: Health Care and Referral of Women in Basic Health Facilities* (2013) Indonesian Ministry of Health, World Health Organization, Indonesian Obstetric and Gynaecology Association, Indonesian Midwifery Association) [41]

Category	Description
Previous pregnancy history:	- stillbirth or neonatal death
	- 3 or more miscarriages
	- baby born < 2500 g or > 4500 g
	- hypertension
	- surgery on reproductive organs
Current pregnancy, maternal:	- maternal age < 16 or > 40
	- Rh(−) blood group
	- hypertension
	- pelvic mass
	- heart disease
	- kidney disease
	- diabetes
	- malaria, tuberculosis
	- HIV, syphilis, other sexually transmitted infection
	- urinary tract infection
	- severe anaemia
	- abuse of drugs or alcohol
	- upper arm circumference < 23.5 cm
	- height < 145 cm
	- weight gain < 1 kg or > 2 kg per month or not appropriate for BMI
	- psychiatric condition
	- any other health condition which can negatively affect pregnancy
Current pregnancy, fetal:	- fundal height not appropriate with gestation
	- multiples
	- small for gestational age
	- malpresentation
Special considerations:	- family problems
	- psychosocial issues
	- violence in the home
	- financial issues
	- other psycho-socio-economic issue
Pregnancy with condition which requires emergency referral	- bleeding
	- preeclampsia and eclampsia
	- prelabour rupture of membranes
	- fetal distress
	- other condition which is life-threatening to mother and baby

### Quality assessment

Each study included was assigned a quality grade based on strengths and limitations and risk of bias, using the Effective Public Health Practice Project (EPHPP) Quality Assessment Tool [42]. This tool was developed to enable assessment of health studies including observational studies which makes it ideal for this topic where randomised control trials are not feasible. Nine components of each study are given a grade according to standardised criteria. These components are sample selection, study design, identification and management of confounders, blinding, data collection methods, and participant withdrawals and dropouts. The combination of ratings for each component is used to create an overall study rating of weak, moderate or strong.

### Data extraction

Citations for each database searched were imported into reference software (Zotero) which was then used to sort and exclude articles by title and abstract. Crude data were extracted by two authors and entered directly into Microsoft excel to create a comparison table. The data were clearly too different to attempt meta-analyses.

## Results

**Fig. 1:**
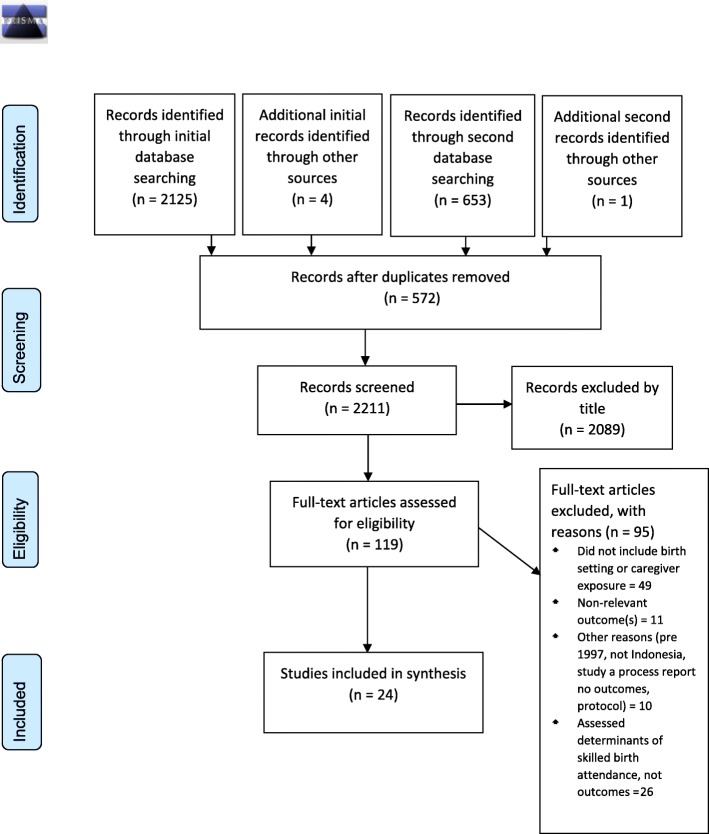
PRISMA Flow Diagram; Schematic presentation of the PRISMA 2009 flow diagram to select and include studies

An initial search in 2016 revealed 2125 studies and four were identified through references of key documents, 407 of which were removed as duplicates. A second search to update the review was conducted in 2018 and identified 653 articles and an additional one was found through relevant bibliographies. The resulting studies were screened by title and abstract and 119 were considered to potentially meet the inclusion criteria and were assessed by full text, with 24 being included in the final review. These studies are listed in Additional file 1: Appendix 1 Table of characteristics of included studies. Reasons for exclusion are listed in Fig. 1: Prisma Flow Chart.

Assessment of quality and risk of bias in the studies was undertaken by one author (KH) using the Effective Public Health Practise Project Quality Rating Tool [42]. Only one study in the final selection was assessed as strong; 11 were given a moderate rating and 12 were given a weak rating.

The 24 studies included represent the breadth of literature which considers the impact of birth caregiver and birth setting on birth outcomes in Indonesia (Table 2). Of the broad range of outcomes in the search strategy, four are represented in the included articles. These are the primary outcomes of neonatal mortality (including early, late, first day, first month and first 90 days) and maternal mortality (and near miss). Secondary outcomes reported in the papers are caesarean section and breastfeeding. Five of the articles encompass only birth setting, including one which compares home birth with facility birth, while the other four compare private and public hospital outcomes. Six of the articles look at the impact of caregiver and not birth setting while 13 include both exposures. Birth setting and caregiver are variously categorised as home birth vs facility birth, private vs public hospital and broader categories (such as clinic, midwifery practice), as well as SBA vs no SBA and by specific caregiver (midwife, obstetrician, traditional birth attendant, nurse etc).

**Table 2:** Relevant exposures and outcomes covered by included papers

		Outcomes (number of risk factors in brackets)			
		Neonatal mortality	Maternal mortality (and near miss)	Caesarean Section	Breastfeeding (including exclusive and early initiation)
Exposures	Setting				
	· Home birth vs facility birth				Yulidasari 2017 (0)
	· Private vs public facilities		Adisasmita et al. (7)	Andayasari et al. 2015 (1) Pristya et al. 2008 (2) Sepehri & Guliani 2017 (2)	
	Caregiver	Stiyaningsih & Wicaksono 2017 (1) SUMMIT Study Group 2008 (5)	Prasetyo, 2018 (0) Ronsmans et al. 2009 (0) Scott 2013 (1) Supratikto 2002 (0)		
	Both setting and caregiver	Abdullah et al. 2016 (7) Belizzi 2017 (1) Dibley et al. 2012 (2) Fort et al. 2008 (1) Hatt et al. 2009 (4) Shrestha 2010 (0) Sutan & Berkat 2014 (3) Titaley, Dibley & Roberts 2012 (2) Titaley et 2010 (2) Titaley & Dibley 2012 (2)			Agushybana 2018 (1) Sari 2016 (1) Titaley et al. 2014 (2)

Of the 24 studies, five (21%) include no measure of risk of complications at birth as defined in Table 1. Another 58% include one or two risk factors, with the most common being maternal age followed by size of the neonate (as proxy for small for gestational age/measurements not congruent with gestation). Two studies include seven risk factors, the highest number considered.

**Fig. 2:**
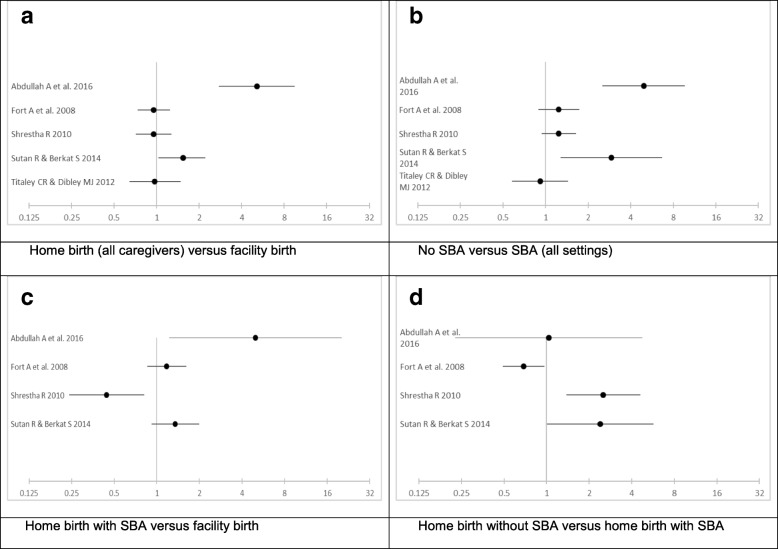
Crude ORs from studies reporting neonatal mortality by birth setting and caregiver; 2a. (top left) Home birth (with and without SBA) versus facility birth, 2b. (top right) Birth with versus without SBA (all settings), 2c. (bottom left) Home birth with SBA versus facility birth (presuming SBA), 2c. (bottom right) Home birth with SBA versus home birth without SBA. *Neonatal mortality was defined as: 0–28 days Abdullah A* et al. *2016; 0–31 days Titaley & Dibley 2012; 0–30 days other studies. X-axis represents odds ratios on log-scale*

The studies are varied in their methodology, though 13 of the 24 sourced their data from the Indonesian Demographic Health Survey (IDHS). Neonatal mortality is the most common outcome and crude results were able to be extracted and presented in Fig. 2. The diversity in categorisation, use of the same dataset and the varying analytical methods made any meta-analysis impossible. The relevant findings of all the included studies are summarised below in a narrative style under the outcomes subheadings.

### Neonatal mortality

Twelve studies report neonatal mortality as their outcome. Two include only caregiver as their exposure while the rest consider both caregiver and birth setting. Two of the studies find that SBA is associated with decreased risk of neonatal death [43, 44], and ten found no statistically significant difference [45–54]. One study reported that facility birth was associated with a decrease in neonatal mortality compared with home birth, two found the opposite and the rest found no statistically significant association. Eight of these studies used IDHS data and eleven considered at least one antenatal risk factor for complications at birth though only nine reported whether or not the risk factors were associated with increased mortality. All nine found that at least one of the reported risk factors was associated with increased risk of neonatal mortality.

Figure 2 shows crude neonatal mortality odds ratios that were able to be calculated from papers which published the data. During extrapolation, it was presumed that all births without SBA occurred at home and therefore subtracting the number of non-SBA births from the number of all home births resulted in the number of home births with SBA. These extrapolations show that there is a difference when comparing all homebirth or homebirth with a SBA to facility birth. They also highlight the lack of consistency reported by the included studies over whether SBA and facility birth improve outcomes or not. This illustrates the importance of an accurate consideration of risk status and other variables which may result in increased consistency.

Interestingly, the one study that did find a significant association between birth at home and neonatal mortality [53] is a case-control study of low birth weight babies. This study controlled for seven pregnancy risk factors, the equal highest number of any study. However, included very few home births with skilled attendance (*n* = 9) making it difficult to interpret whether the increase in mortality was associated with home birth or a lack of SBA.

Titaley, Dibley and Roberts [49] highlighted the difficulty in interpretation when segregating their findings by area. The authors reported that in rural areas, births attended by trained health professionals, regardless of setting were actually associated with an *increased* risk of neonatal mortality. While in urban areas, birthing in a health facility resulted in a protective factor if the mother had complications at birth. This could be pointing to a tendency for women in rural areas to seek help too late for their babies to be saved, or a tendency for only high risk women birthing in rural areas to have health professionals present.

### Maternal mortality

Five studies report on maternal mortality outcomes. One compares private and public facility birth [55] and the other four studies appraise the association with caregiver at birth but not birth setting [56–59].

Of those comparing caregiver, one found no statistically significant association [56] while two actually found that the presence of an SBA is associated with higher maternal mortality [57, 58]. The final paper did not report any significance testing [59]. These papers argue, however, that this association may be because, in situations where uptake of SBA is low, woman may only be seeking a doctor or midwife when complications or illness arise, perhaps too late to be saved. Ronsmans et al. [57] report that the likelihood of birthing with a health professional is correlated with increasing wealth status, and a decrease in maternal mortality ratio (MMR). The odds of having a health professional present at the time of birth is 1.9 times higher among maternal deaths than survivals, and only 10.3% of women in the poorest wealth quintile birth with a health professional. Scott et al. [58] report increasing maternal mortality with greater distance from a health facility when a health professional is present, but not when no health professional is present for the birth,

Adisasmita et al. [55] reports that a higher percentage of maternal deaths and near misses occurred in public as opposed to private hospitals (17.3% vs 4.2%, and 1.6% vs 0.1% respectively). Public hospitals also had a higher percentage of women in a critical condition at admission, and a lower percentage of normal births without complications. While no analysis of the statistical significance of these percentages was conducted, this study adds weight to the argument that some women may be seeking care too late to be saved. This is one of the few studies which reported on a significant number of antenatal risks of complication at birth, including maternal age, infection, hypertensive disease, haemorrhage, anemia, malpresentation and “other conditions”. Without tests of significance, however, it cannot be deduced whether these risk factors were significantly associated with mortality or near miss.

#### Caesarean section

Three studies assessed caesarean section and all compared private with public hospitals [60–62]. Two reported a greater per cent of caesarean sections in private hospitals compared with public, while the other reported the opposite [62]. Two contradictory studies used DHS data, one Indonesia-wide, while Pristya included only urban areas which is where the higher public hospital caesarean section numbers were found. This suggests that rural public hospitals may have higher caesarean section rates, supporting the previous theories of women in these areas seeking help when complications (and therefore need for intervention in the form of caesarean section) is required. The third study, however, was from Jakarta based hospital data, an urban area. Two of the studies reported on antenatal risk factors and both found them to be associated with increased risk of caesarean section, again highlighting the importance of considering antenatal variables with birth outcomes.

### Breastfeeding

Four studies compared breastfeeding outcomes; three were concerned with exclusive breastfeeding [63–65] and one with delayed initiation [66]. No association was found between birth setting and risk of exclusive breastfeeding in any of the three studies. Titaley et al. [66] found that babies born in government owned facilities, outside of health facilities and via caesarean section had a reduced odds of initiating breastfeeding within 1 hour of birth than those born vaginally in private health facilities.

### Risk status

It was impossible to determine from the included studies outcomes specifically for low risk women. Five of the 24 studies included no risk factors, another 14 adjusted for either one or two risk factors and the other five considered between three and seven risk factors. The risk factors included were most commonly maternal age and neonatal size but other factors such as anemia, history of previous pregnancies or twins were sometimes considered (see appendix for details). However, none could be said to have been comprehensive when compared with the list in Table 1 and they made no distinctive risk grouping making it impossible to compare birth outcomes in different settings for only low risk women. That means that no conclusion can be drawn about the safety of attended home birth for low risk women in Indonesia.

Of those which did include antenatal risk factors for complications at birth, nearly all found one or more of them to be association with the outcomes assessed. This shows the impact of risk factors on birth outcomes and the importance of their consideration when determining the impact of birth settings and caregivers on neonatal and maternal mortality in particular.

## Discussion

While the outcomes and findings of this review included studies are diverse, they paint an overall picture that the impact of birth setting and caregiver on birth outcomes is not as clear as many policies imply. Most of the included studies find that birth setting and caregiver are not statistically significant in their associations with neonatal mortality or breastfeeding. Two studies found an increased risk of maternal mortality with SBA as opposed to no SBA, but both found this among populations where uptake was low (more remote and poorer women). SBA was found to improve neonatal mortality among just two studies yet also two studies found an increase in risk of neonatal death among facility births when compared with home births. These findings are particularly interesting given that a key message from the World Health Organization is that skilled care at birth is essential to save maternal and neonatal lives.

The poor clarity of maternal risk status in the included studies suggests that assumptions were made by the authors about their outcomes. For example, where studies found higher mortality rates in hospitals or when births were attended by trained health professionals, compared with at home without trained assistance, the authors concluded that women were probably seeking care when they became ill and could be reaching assistance too late to be saved [43, 49, 55, 57]. This may well be the case, however it is also true that if a maternity system is working well then women who develop a risk factor for an adverse outcome should be referred to and receive back-up obstetric care in hospitals and therefore any unfortunate ‘near misses’, maternal or neonatal deaths should only be occurring in hospitals.

Without any clear pregnancy and birth risk assessment, it is impossible to conclude if the appropriate women are being transferred to hospital, arriving too late to be saved, or if these deaths are due to some other factor. For example, perhaps the care being provided by health professionals in hospitals is leading to iatrogenic effects and poorer outcomes, or women who live further away from hospitals may have a poorer underlying health status and therefore more prone to adverse outcomes outside of hospitals.

The issue of risk status and other confounding factors likely contribute to the inconsistent, and sometimes contradictory findings of the 24 included studies assessing the impact of birth carers and settings. Ideally, any study attempting to ascertain the impact of birth setting or caregiver needs to take into consideration the appropriateness of the setting and carer for that woman, i.e. risk status at the point of labour onset and ongoing, as well as the woman’s preferences and the options available to her. Assessing maternal intentions and whether the actual place of birth was the intended place of birth allows for accurate assessment of the impact of transfers in labour, and the effectiveness of the health system.

This finding that risk of complications is not adequately considered is reflected in previous systematic reviews. Scott and Ronsmans [67] conducted a literature review in 2009 of the relationship between maternal mortality and birth with a health professional. They included ten observational studies which were of poor quality, did not control for confounding factors well and found overall that birth with a health professional did not improve outcomes, and in some cases made them worse.

Similarly, Tura et al. [68] conducted a 2013 systematic review and meta-analysis into the effect of health facility delivery on neonatal mortality. They included 19 studies from low and middle income countries and found that health facility delivery could reduce neonatal mortality by 29%. However, the only adjusting for risk that they conducted was to exclude studies that compared planned hospital births with planned home births, because they presumed that the planned hospital births would be high risk. In fact, they excluded studies for this reason which were specifically comparing only low risk women in both groups [10, 11].

The studies included in this review were all but one given a quality rating of weak or moderate using the EPHPP tool. This is in part due to the difficulty in acquiring data which allow important potential confounders such as risk status to be taken into consideration. Data is frequently sourced from hospital records, excluding birth in communities, or from survey data such as the IDHS, which is open to recall bias and does not ask for enough details to permit accurate assessment.

### Limitations

While language was not specifically restricted in the search strategy, terminology in Indonesian language was not searched, therefore some articles may not have been found. It is standard practice, however, for Indonesian research to provide an abstract translated into English, which should have been found in the search. Another limitation was that only one author undertook review of the quality of studies using the EPHPP tool.

## Conclusions

Studies which attempt to assess outcomes of birth by setting and caregiver in Indonesia and other low resource countries are conflicting in their results, making it difficult to draw conclusions. The studies fail to stratify by or adequately control for risk status of women, skill of caregiver and intended place of birth. Without these controls in place it is impossible to comment on the direct impact of a program which supports attended homebirth outside of high income countries. This ambiguity has implications on maternity health systems development, policies and funding priorities which impact women’s and babies’ health outcomes.

## Additional files

Additional file 1: **Appendix 1.** Table of characteristics of included studies. (DOCX 43 kb)

Additional file 2: **Appendix 2.** Search terms. (DOCX 51 kb)

## Abbreviations

ANC: antenatal care
EPHPP: Effective Public Health Practice Project
IDHS: Indonesian Demographic Health Survey
MMN: Multiple micronutrient
MMR: Maternal Mortality Ratio
SBA: Skilled Birth Attendant
TBA: traditional birth attendant
