# Supplementary material for: Outcomes by birth setting and caregiver for low risk women in Indonesia: a systematic literature review
Source: Reprod Health. 2019 May 28;16:67. doi: 10.1186/s12978-019-0724-7 (PMC6540424; doi:10.1186/s12978-019-0724-7)
Supplement: Supplementary file 1 — Appendix 1. Table of characteristics of included studies. (DOCX 43 kb) [file 12978_2019_724_MOESM1_ESM.docx]

| **Article (author, year)** | **EPHPP quality rating** | **Study type** | **Data source, location and year/s** | **Sample size, population** | **Relevant exposure/s and outcome/s** | **Measure/s of risk (as per table 1)** | **Confounders/other variables** | **Relevant Results** |
| --- | --- | --- | --- | --- | --- | --- | --- | --- |
| Abdullah A et al. 2016 | Weak | Retrospective matched case-control | Puskesmas (health clinic) death reports health records from Nusa Tenggara Timur, 2013 | 154 cases of neonatal deaths, 308 matched live birth controls | Exposure: Birth setting (home/healthcare facility); Birth carer (TBA/nurse/midwife or doctor)  Outcome: Neonatal mortality (0-28 days) | Previous pregnancy: history of previous pregnancies (proxy for history of 3 or more miscarriages)  Current pregnancy, maternal: Illness during pregnancy (interview with mother), complications during pregnancy (antenatal record), haemoglobin (proxy for anemia from antenatal record), age, risk status during pregnancy.  Current pregnancy, fetal: birthweight (proxy for small for gestational age) | Use of iron tablets in pregnancy, maternal knowledge of whether pregnancy was high risk, maternal knowledge of danger signs of pregnancy and birth, mother informed of due date, mother knew danger signs of newborn, complications during birth, age at marriage, birth location and assistant, neonatal sex, neonatal problem requiring visit to healthcare provider, Apgar score, early initiation of breastfeeding, kangaroo method of care, distance and travel time from village to health centre, highest level of parents education, economic status. | In the multivariate analysis home birth was found to be significantly associated with neonatal death when compared to healthcare facility birth (OR 8.65, 95% CI 1.11-67.49, p<0.05). Presuming all of the births attended by TBAs were at home, only 9 home births with skilled attendants were included; 6 cases and 3 controls. Birth carer was not included in the final analysis.  Complications during pregnancy were significantly associated with neonatal death in the univariate analysis, but not in the multivariate (OR 2.84, 85% CI 0.39-20.68) |
| Adisasmita A et al. 2008 [47] | Weak | Retrospective cross sectional, prevalence | Health records from four public and private hospitals, West Java, Nov 2003-Oct 2004 | 5,669 pregnancy related admissions | Exposure: Birth setting (public hospital/private hospital).  Outcome: maternal death and serious morbidity (near miss).  Secondary outcome: normal birth | Current pregnancy maternal: age, infection, hypertensive disease, antepartum haemorrhage, anemia, malpresentation, other conditios | Parity, state (critical or not) at admission. | When compared to private hospitals, public hospitals had higher percentages of maternal death and near miss (17.3% vs 4.2%, and 1.6% vs 0.1% respectively), women critical at admission and lower percentages of normal births without complications (8.7% vs 34%). No analysis of significance was conducted. |
| Agushybana, F et al. 2018 | Weak | Retrospective cross-sectional survey | Indonesia-wide DHS 2012 | 1508 women who gave birth over the previous two years, were currently breastfeeding and infants 0-5 months living with mother | Exposure: Birth setting (home, hospital, health centre, health post, maternity hospital), birth carer (GP, obstetrician, midwife, nurse, other)  Secondary outcome: exclusive breastfeeding | Current pregnancy, maternal: age | Education, occupation, household wealth index, region of Indonesia, caesarean section, early breastfeeding initiation, postnatal health checkup, access of information (newspaper, radio, television). | In the multivariate analysis, no birth setting or caregiver were statistically significant with exclusive breastfeeding. |
| Andayasari L et al. 2015 [48] (Indonesian language) | Weak | Retrospective cross sectional, prevalence | Health records from one public and one private hospital, Jakarta, 2011 | 2479 births | Exposure: birth setting (private and public hospital)  Secondary outcome: Caesarean section | Current pregnancy, maternal: age | Education, employment, insurance status, antenatal care, hospital room class. | When compared with the private hospital, the public hospital had a lower caesarean section rate (56.5% vs 62.7%). No analysis of significance was conducted. |
| Bellizzi S et al. 2017 | Weak | Retrospective cross-sectional survey | Indonesia-wide DHS 2012 (with DHS from eight other low and middle-income countries) | 15068 most recent live births from ever-married women age 15-49 years | Exposure: Birth setting and carer composite (home, home with SBA, health facility)  Outcome: Neonatal deaths (early, not defined) | Current pregnancy, maternal: age | Labour complications (high fever, convulsions, vaginal bleeding, prolonged labour), socio-economic status, birth order and spacing | In multivariate analysis, no statistically significant difference was found in early newborn mortality between facility birth and home birth with and without SBA. (Non-SBA home birth vs SBA home birth OR 0.9, 95% CI 0.4-1.6; SBA home birth vs health facility OR 0.9, 95% CI 0.5-1.6; non-SBA home birth vs health facility birth OR 0.9, 95% CI 0.5-1.6) in the Indonesian subset. |
| Dibley MJ et al. 2012 [38] | Moderate | Retrospective cross-sectional survey | Indonesia-wide DHS 1994, 1997, 2002–2003, and 2007 | 52,917 singleton live-born infants | Exposure: Birth carer (TBA/other untrained/none, health professional), birth setting (institutional, home) – not primary exposures for this study.  Outcome: Neonatal mortality. | Current pregnancy, maternal: age,  current pregnancy, fetal: birth size and duration of pregnancy (composite, proxy for fundal height appropriate with gestation/small for gestational age) | Urban or rural areas, the average cluster coverage of Bacillus Calmette-Guérin vaccine against tuberculosis vaccination or average cluster coverage of measles vaccination, maternal level of attained education, maternal marital status, parental occupation, and household wealth index, child sex, birth rank and birth interval, the mother's desire for the pregnancy, any reported delivery complications (including prolonged labor, excessive vaginal bleeding, fever or foul smelling, or convulsions), breastfed, antenatal care mode of delivery, iron and folic acid supplementation - primary exposure for this study. | Attendance of a health professional at birth resulted in a non-statistically significant reduction in neonatal mortality compared with a non-trained attendant (HR 0.81, 95% CI 0.66-1.00). Birth setting was not included in the final analysis as it was found to be non-significant at p=0.05 in an earlier stepped model. A third of women birthed in a facility, 60% with a trained birth attendant. |
| Fort A et al. 2008 [41] | Moderate | Retrospective cross-sectional survey | Indonesia-wide DHS survey 2003 (with Bangladesh 2004, India 2005-06, Nepal 2006 and Philippines  2003). | Not reported | Exposure: Birth carer (skilled, non-skilled), birth setting (institutional, non-institutional)  Outcome: Neonatal mortality (0-30 days) | Current pregnancy, fetal: newborn size (proxy for small for gestational age) | Urban/rural, education, wealth, baby sex, birth order and interval, any ANC, informed of complications, given iron supplements, tetanus injection, use of disposal delivery kit, clean instrument used to cut cord, anything on stump, caesarean birth, long labour, excessive bleeding, baby’s hands and feet came first, fever/convulsions, postpartum care, early breastfeeding, wrapped immediately after birth, timing of bath, maternal examination, family planning advice, breastfeeding advice, baby care advice, newborn check-up | Mother’s reported size of baby as small or very small at birth (the only risk factor reported on) associated with higher neonatal mortality in Indonesia (NMR per 1000 live births for small or very small baby at birth 39, 95% CI 25-54; average or larger 12, 95% CI 8-15) as well as in the other countries. No association found in Indonesia with birth setting (NMR for institutional birth 16, 95% CI 10-22; non-institutional birth 15, 95% CI 11-20) or skilled attendant at birth (NMR for doctor, nurse, midwife 11, 95% CI 7-14; for ‘other’ attendant 13, 95% CI 7-19) or the other countries reported on. |
| Hatt L et al. 2009 [42] | Moderate | Retrospective cross-sectional survey | Pooled Indonesia-wide DHS 1991, 1994, 1997 and 2002/3 | 65 921 live births | Exposure: Birth setting (home including midwife’s home, public and private facility) and birth carer (professional attendance or not)  Outcome: First day and early neonatal mortality (0-6 days). | Previous pregnancy history: history early neonatal death  Current pregnancy, maternal: age,  Current pregnancy, fetal: multiple pregnancy, baby size at birth (proxy for small for gestational age) | Wealth, education, rural/urban, parity, birth interval and baby sex | There was a decrease in neonatal deaths across the time frame 1986-2002. The adjusted OR for birth setting and professional attendance was only significant in this trend for early neonatal deaths at home births with no professional attendance (OR per year 0.96, 95% CI 0.93-0.99). Of the risk factors included in the logistic regression, maternal age (aOR 0.81, 95% CI 0.72-0.91), multiple pregnancy (aOR 4.21, 95% CI 2.82-6.28) and smaller than average (aOR 3.25, 95% CI 1.86-5.66) or very small baby (aOR 15.60, 95% CI 8.69-28.00) were significantly associated with early neonatal deaths. Multiple pregnancy (aOR 3.89, 95% CI 2.36-6.43), smaller than average (aOR 4.01, 95% CI 1.91-8.43) and very small baby (aOR 18.76, 95% CI 8.42-41.80) were also associated with first-day neonatal deaths. |
| Prasetyo, B et al. 2018 | Weak | Retrospective case-control | East Java maternal mortality reports 2015-2016 | 125 maternal deaths in five regencies/cities with highest mortality (case groups) and five regencies/cities with the lowest maternal mortality (controls groups) | Exposure: Birth caregiver (per cent of births covered by SBA in the group),  Outcome: Maternal mortality detection by MMR | None though percentage of high risk pregnancies (not defined) detected in case vs controls is reported. | None, though the percentage of pregnancies with ANC, puerperal health care and obstetric complication management in cases vs controls is given. | 16 of 125 maternal deaths occurred at home, 4 during transfer, the rest in a facility, including 78.4% in a hospital. No difference in SBA coverage in areas with highest compared to lowest MMR (95.54, SD 5.37 vs 95.93, SD 4.55), or in detection of high-risk pregnancy (14.75, SD 4.01 vs 12.98, SD 3.31). |
| Pristya, TYR et al. 2018 | Weak | Retrospective cross-sectional survey | Indonesia-wide DHS 2012 | 5143 women aged 15-49 who lived in urban areas | Exposure: birth setting  Secondary outcome: caesarean section | Current pregnancy, maternal: age,  NB In non-final model ‘medical indication: complication’ is included, but not defined and dropped by the final analysis though it was significant in the univariate. | Antenatal care provider and facility, parity. | Birth in a private facility had 0.4 reduced odds of caesarean section than a public facility (95% CI 0.3-0.5). There was a 23% overall caesarean section rate (greater than national average of 16.8%). The risk factor of maternal age was also associated increased risk of caesarean section (>34yo OR 1.4, 95% CI 1.1-1.9) |
| Ronsmans C et al. 2009 [46] | Moderate | Case-control study and cohort analysis | Maternal death audit and population based survey from two districts of West Java Jan 2004-Dec 2005 | 458 cases, 1234 controls | Exposure: birth caregiver (health professional)  Outcome: maternal mortality | None | Maternal wealth, education, insurance, urban/rural, district and population level midwife density. | Maternal mortality was associated with presence of health professional at birth (crude OR 1.85, 95% CI 1.4-2.5). |
| Sari, Y 2016 | Moderate | Retrospective cross-sectional survey | Indonesia-wide DHS 2012 | 1193 mothers aged 15-49 who had 0-5 month old living babies | Exposure: birth caregiver, birth setting  Secondary outcome: exclusive breastfeeding. | Current pregnancy, maternal: age | Maternal education, paternal education, household wealth index, type of residence, exposure to information source, parity, (in bivariate analysis but not final multivariate analysis), mode of birth, ANC frequency and trained ANC provider | SBA and place of birth not statistically significant for breastfeeding. Caesarean birth negatively correlated with exclusive breastfeeding (OR 1.6 95%CI 1.1-2.5). |
| Scott S et al. 2013 [51] | Moderate | Case-control | Maternal death reports and population based survey from West Java 2004-2005 (uses data reported in Ronsmans et al 2009) as well as Matlab, Bangladesh 1987-2005 | 1234 controls in Indonesia, 53 924 in Bangladesh | Exposure: Birth caregiver  Outcome: Maternal mortality. | Current pregnancy, maternal: age | parity, education, household assets, distance to health facility | Increased maternal mortality was associated with greater distance from health centre when a health professional was present for the birth (aOR for every 2.5km greater distance 1.07, 95% CI 1.02-1.11), but not when no health professional was present (aOR 1.01, 95% CI 0.96-1.07). |
| Sepehri A & Guliani H 2017 | Weak | Retrospective cross-sectional survey | Indonesia wide DHS 2012 with DHS Bangladesh (2011), India (2005/06), Pakistan (2012/13) and Philippines (2013) | 8645 Indonesian women who birthed in facilities | Exposure: Birth setting (government hospital, other public health facility, private hospital/clinic)  Secondary outcome: Caesarean section | Current pregnancy, maternal: age  Current pregnancy, fetal: multiple birth | Parity, education, household wealth, urban/rural, high/low population level of caesarean section per province/state | Odds of caesarean section in private hospital 1.36 (95%CI 1.17-1.58) compared with government. Birthing in a private hospital in a low use area decreased odds compared with medium use area. The risk factor of increasing maternal age was also association with increase odds of caesarean section (OR 1.96, 95% CI 1.38-2.07) |
| Shrestha R 2010 [27] | Moderate | Retrospective cohort survey | Indonesian Family Life Survey (IFLS) 1993, 1997, 2000 | 7,224 households including over 22000 individuals in original cohort from 13 of the 27 provinces of Indonesia | Exposure: Birth setting (home, midwife clinic, hospital, puskesmas (clinic), physician clinic, TBA premises) and caregiver (midwife, TBA, physician, other, nurse)  Outcome: neonatal (first month) mortality | None | Aggregates village midwife prevalence at district level | Neonatal mortality rates highest for those born in hospital (46.9%, SD 9.4 in bidan di desa communities, 37.5%, SD 7.7 in non-bidan di desa communities). Infant mortality rates are lowest for babies born with midwives (16.1%, SD 2.8 in bidan di desa communities and 17.5%, SD 3.4 in non-bidan di desa communities) than physicians, nurses or TBAs. |
| Stiyaningsih H & Wicaksono F 2017 | Weak | Retrospective cross-sectional survey | Indonesia-wide DHS 2012 | 9754 women aged 15-49 who had their last birth in previous 5 years | Exposure: birth caregiver (SBA)  Outcome: neonatal mortality | Current pregnancy, maternal: age | birth interval, sex of child, ANC, women’s empowerment (composite index of variables: participation in economic decision-making, health decision-making and autonomy in mobility) | SBA associated with 0.5 odds (95%CI 0.50-0.93) neonatal mortality compared with no SBA. The risk factor of age was also significant with those 20-35 years old having 0.47 odds of experiencing infant mortality (95% CI 0.35-0.62) compared with those older and younger.  High level of women’s empowerment has 0.14 reduced odds of experiencing neonatal mortality (95%CI 0.05-0.38) compared with low empowerment. |
| Supplementation with Multiple Micronutrients Intervention Trial (SUMMIT) Study Group et al. 2008 [44] | Strong | Double-blind cluster-randomised trial | Lombok July 1 2001 to April 1 2004 | 31 290 pregnant women received either iron and folic acid (IFA) supplementation (n=15 486), or multiple micro-nutrient (MMN) supplementation (n=15 804). | Exposure: Birth caregiver  Outome: Early neonatal mortality (0-7 days), late neonatal mortality (8-28 days), fetal loss, | Previous pregnancy history: previous child death  Current pregnancy, maternal: age, morbidity history, mid upper arm circumference, haemoglobin (for a subset)  Current pregnancy, fetal: low birthweight | Parity, dietary practices, socioeconomic status, gestation at birth and service post of midwife. | Having a trained attendant at birth was non-significantly associated with neonatal death. Infants of women with trained attendants experienced 20% fewer deaths if the mother received MMN, this was not significant with untrained birth attendants. |
| Supratikto G et al. 2002 [52] | Weak | Maternal and perinatal mortality audit and verbal autopsy | South Kalimantan, 1994-1999 | 130 maternal deaths | Exposure: birth caregiver (family member, TBA, midwife, doctor, other)  Outcome: maternal mortality |  | Contributing factors including delays in decision making, delays in receiving care and quality of care received. | Most maternal deaths occurred with a TBA being the highest level of birth attendant seen (35.4%) compared with midwife (24.6%), doctor (16.9%) or other/unknown (23.1%). 69.2% of maternal deaths occurred outside of a health facility. No significance analysis was done, and no comparison group is available. |
| Sutan R & Berkat S 2014 [49] | Weak | Unmatched case control | Neonatal death records and child health clinic records, Aceh 2010-2012 | 250 neonatal deaths, 250 live births of low birth weight babies | Exposure: birth setting (health facility, non-health facility) and caregiver (TBA, trained personnel)  Outcome: neonatal death. | Current pregnancy, maternal: age, illness  Current pregnancy, neonatal: birth weight and referral for LBW (proxy for small for gestational age) | Sex, gestational age, maternal death, birth interval, parity, education level, >4 antenatal visits, caesarean section/vaginal birth, appropriate neonatal visits with health professional, time of first and frequency of bathing, kangaroo mother care, use of warm bottle pack, use of lamp bulb, ‘didaring’ (sitting with baby by fire), umbilical cord care, hand washing before touching baby, initiation of breastfeeding <1 hour after birth, discarding colostrum, exclusive breastfeeding | In the regression analysis of neonatal and maternal factors, birth setting and birth attendant were not significantly associated with neonatal mortality among low birth weight babies. Among the risk factors included, lower birth weight (<1500g aOR 17.84, 95% CI 6.20-51.35) and maternal illness (aOR 1.87, 95% Ci 1.06-3.30) were significantly associated with neonatal mortality. |
| Titaley CR, Dibley MJ & Roberts CL 2012 [43] | Moderate | Retrospective cross-sectional survey | Indonesia-wide DHS 1994, 1997, 2002/3 and 2007, pooled | 52 917 singleton live-born infants | Exposure: Birth setting and caregiver (untrained attendant at home, trained attendant at home, public hospital, private hospital, other public birthing centre, other private birthing centre)  Outcome: Neonatal mortality (0-7 days) | Current pregnancy, maternal: age  Current pregnancy, fetal: combined birth size and timing (proxy for small for gestational age) | Household wealth index, combined birth rank and interval, delivery complication, community region and average paternal schooling, maternal marital status, paternal education, parental occupation, desire for pregnancy, baby sex, use of antenatal care and mode of birth | In the multivariate analysis, no reduction in risk of death was seen between births at home with untrained attendants and trained attendants (aHR 1.24, 95% CI 0.82-1.87). There was, however, an increased risk in public hospitals compared to other settings (aHR 2.65, 95%CI 1.48-4.75). Of the risk factors, smaller than average was associated with greater risk of neonatal death. |
| Titaley CR et al. 2014 [53] | Moderate | Retrospective cross-sectional survey | Indonesia-wide DHS 2002/3 and 2007, pooled | 12,191 singleton live-born children 0-23 months old | Exposure: Birth setting (private health facility, government-owned health facility, non-health facility) and caregiver (non/untrained, trained delivery attendant)  Outcome: Breastfeeding (delayed initiation and non-exclusive). | Current pregnancy, maternal: age  Current pregnancy, fetal: size (proxy for small for gestational age) | ANC, mode of birth, desire for pregnancy, birth rank and interval, birth complication, duration of pregnancy, region, type of residence, household wealth index, maternal education, paternal education, parental occupation, maternal marital status, maternal final say on healthcare, frequency of reading newspaper, listening to radio and watching television. | In the multivariate analysis, birth in a government owned (aOR 1.38, 95% CI 1.08-1.76) and non-health facility (aOR 1.20, 95% CI 1.00-1.43) were associated with delayed initiation of breastfeeding when compared with a private health facility, but trained birth attendant was not. |
| Titaley CR et al. 2010 [39] | Moderate | Retrospective cross-sectional survey | Indonesia-wide DHS 1994, 1997 and 2002/3, pooled | 40 576 singleton live infants (the most recent child born to mothers in the 5 years previous to each survey). | Exposure: Birth setting (health facility, non-health facility) and caregiver (untrained/none, trained attendant)  Outcome: Early neonatal death (<7 days old) | Current pregnancy, maternal: age  Current pregnancy, fetal: birth size and timing (proxy for small for gestational age) | ANC, tetanus toxoid vaccination, iron folic acid supplementation, mode of birth, year of birth, region of residence, paternal education and occupation status, maternal marital status, household wealth index, sex of the child, birth rank and interval, desire for pregnancy, delivery complications. | In multivariate analysis, neither birth setting or birth caregiver were significantly associated with neonatal mortality. Of the risk factors, age was not significant, while smaller than average size was. |
| Titaley CR & Dibley MJ 2012 [40] | Moderate | Retrospective cross-sectional survey | Indonesia-wide DHS 2002/3 and 2007, pooled | 26 591 singleton live infants (the most recent child born to mothers in the 5 years previous to each survey). | Exposure: Birth setting and caregiver  Outcome: Early neonatal mortality (<7 days) and all neonatal mortality (<1 month) | Current pregnancy, maternal: age  Current pregnancy, fetal: birth size and timing (proxy for small for gestational age) | Year of survey, region, type of  residence (urban/rural), average paternal year of  schooling in the cluster, maternal year of education, household wealth index, child sex, combined birth rank and interval, maternal  desire for pregnancy, reported delivery complications  (including prolonged labour, excessive vaginal bleeding,  fever/foul smelling and convulsions), antenatal  iron/folic acid supplements, postnatal care, use  of antenatal care, mode of  birth,  duration of the recall period between date of childbirth  and interview, and economic status. | Birth in a health facility and birth with a trained attendant did not significantly affect early neonatal mortality. Both risk factors were significantly associated with early neonatal death (increased maternal age aHR 1.05, 95%CI 1.01-1.09; smaller than average birth size aHR 4.33, 95%CI 2.64-7.12). |
| Yulidasari F et al 2017 | Weak | Observational analytical case-control | Sungai Ulin Public Health Center health records, South Kalimantan 2016 | 132 mothers with healthy children aged 6-12 months | Exposure: health facility birth  Secondary outcome: exclusive breastfeeding | None | Husband support | Birth setting not statistically significant (included just 2 women who did not birth in a health facility). Husband support results in increased odds of exclusive breastfeeding (OR 6.75 95%CI 1.796-25.370) |
