# Supplementary material for: Outcomes by birth setting and caregiver for low risk women in Indonesia: a systematic literature review
Source: Reprod Health. 2019 May 28;16:67. doi: 10.1186/s12978-019-0724-7 (PMC6540424; doi:10.1186/s12978-019-0724-7)
Supplement: Supplementary file 2 — Appendix 2. Search terms. (DOCX 51 kb) [file 12978_2019_724_MOESM2_ESM.docx]

## **Appendix 2:** Search terms

Keywords:

- Birth setting
  - keywords
    - “Place of (birth OR delivery)” OR “birthplace” or “birth place” OR Childbirth OR “(Birth OR delivery) setting” OR “alternative birth methods” AND…
    - “Birth centre” OR “birthing centre” OR “birth center” or “birthing center”
    - (Birth OR delivery) suite
    - “Home birth” OR “home childbirth” OR “homebirth” OR “out of hospital birth” OR “out-of-hospital birth”
    - Traditional birth attendan*
    - Midwi* OR “midwife*-led” or “nurse-midwife*”
    - “Obstetric*” OR “hospital birth” OR “obstetric unit” OR “delivery, obstetric” OR “medical birth”
  - MeSH:
    - Parturition
      - **Home childbirth**
      - Natural childbirth
    - Pregnancy outcome
    - Delivery, obstetric
    - Midwifery
- AND (Indonesia OR java OR Kalimantan OR Sulawesi OR Surabaya OR papua OR bali)

Limit to human and female. Do not limit language.
